# Frailty and inflammatory markers in older adults with cancer

**DOI:** 10.18632/aging.101162

**Published:** 2017-03-08

**Authors:** Tomohiro F. Nishijima, Allison M. Deal, Grant R. Williams, Emily J. Guerard, Kirsten A. Nyrop, Hyman B. Muss

**Affiliations:** ^1^ UNC Lineberger Comprehensive Cancer Center, Chapel Hill, NC 27599, USA; ^2^ University of Alabama at Birmingham, Division of Hematology and Oncology, Birmingham, AL 35294, USA; ^3^ University of Wisconsin, Division of Hematology and Oncology, Madison, WI 53792-5669, USA

**Keywords:** frailty, inflammatory markers, lymphocyte monocyte ratio (LMR), neutrophil lymphocyte ratio (NLR), older adults with cancer

## Abstract

We examined the associations between frailty and inflammatory markers, in particular neutrophil lymphocyte ratio (NLR), in elderly cancer patients. We conducted cross-sectional analyses of data derived from the Carolina Seniors Registry (CSR), a database of geriatric assessments (GA) in older adults (≧65 years) with cancer. We included patients in the CSR who had a GA and complete blood count test before initiation of therapy. The primary outcome was frailty, determined using the 36-item Carolina Frailty Index (CFI). In our sample of 133 patients, the median age was 74, and 54% were robust, 22% were pre-frail, and 24% were frail. There was a significant positive correlation between CFI and NLR (r = 0.22, p = 0.025). In multivariable analysis, patients in the top tertile of NLR had an odds ratio of 3.8 (95% CI = 1.1-12.8) for frail/pre-frail status, adjusting for age, sex, race, education level, marital status, cancer type and stage. In bivariable analyses, higher NLR was associated with lower instrumental activity of daily living (IADL) score (p = 0.040) and prolonged timed up and go (p = 0.016). This study suggests an association between frailty and inflammation in older adults with cancer.

## INTRODUCTION

Frailty is characterized by a decline in function and physiologic reserve across multiple organ systems [1]. As a result, it is associated with high vulnerability to stress and increased risk of adverse health outcomes, such as disability, dependency, and mortality [1]. In geriatrics, two commonly used definitions of frailty use approaches developed by Fried and Rockwood. Fried's model defines frailty as the presence of ≥ three of five specific criteria: weight loss, exhaustion, weak grip strength, slow walking speed, and low physical activity [2]. Rockwood's approach focuses on the cumulative impact of a patient's clinical deficits identified by chronic diseases, signs, symptoms, and abnormal test results [3]. These measures have been validated against adverse outcomes including morbidity and mortality in large epidemiological studies [4-6]. In geriatric oncology, the most well-known frailty screening tool is the Balducci criteria consisting of four items (age, activity of daily living (ADL), comorbidity, and geriatric syndromes) [7], which is based on the definition proposed by Winograd et al. [8]. Applying the frailty index to oncology, Guerard et al. recently developed and validated the 36-item Carolina Frailty Index (CFI) for older adults with cancer using the deficit accumulation approach [9, 10]. The CFI uses measures from a cancer-specific geriatric assessment (GA) [11] to calculate a patient's total number of deficits. The CFI was prognostic of all-cause mortality in a diverse group of older adults with cancer [10].

Associations between frailty and markers of inflammation have been described in the general population of older adults. For example, high total white blood cell (WBC) counts have been associated with an increased prevalence of frailty in community-dwelling older women [12]. An increase in neutrophil and monocyte counts was also positively associated with frailty in the same cohort [13]. Furthermore, in a cross- sectional study of older people, higher neutrophil and lower lymphocyte counts were associated with low physical activity while lower lymphocyte counts were correlated with poor muscular strength [14]. In cancer patients, it has been shown that increased WBC, neutrophil and monocyte counts and decreased lymphocyte counts are associated with higher mortality [15-17].

## RESULTS

### Patients' characteristics

Participants' characteristics are shown in Table 1. Of 133 evaluable patients, median age was 74 years (range 65-92), 88% white, 80% female, 89% had at least a high school education, and 57% were married. The most common type of cancer was breast cancer (59%) and most (73%) had localized cancer. Outcome measures and inflammatory markers were summarized in Table 2. Mean of CFI for the study population was 0.22, with 54% classified as robust, 22% as pre-frail, and 24% as frail.

**Table 1:** Patients' characteristics

Patients' Characteristics (N=133)		
Characteristic	No. of Patients	% Patients
Age, years		
65–69	32	24.1%
70–74	39	29.3%
75–80	28	21.1%
80–85	21	15.8%
>85	13	9.8%
Sex		
Male	27	20.3%
Female	106	79.7%
Race		
White	117	88.0%
Non-White	16	12.0%
Educational level		
Less than high school	13	10.7%
High school graduate	49	40.2%
Associate/Bachelors	35	28.7%
Advanced degree	25	20.5%
Marital status		
Married	69	57.0%
Divorced	12	9.9%
Widowed	36	29.8%
Single	4	3.3%
Cancer type		
Breast	79	59.4%
Lung	14	10.5%
Genitourinary	9	6.8%
Gastrointestinal	8	6.0%
Other	23	17.3%
Cancer stage		
I	36	27.1%
II	42	31.6%
III	19	14.3%
IV	36	27.1%
Physician-rated KPS		
<60 (%)	7	5.3%
60-80 (%)	22	16.5%
80-100 (%)	104	78.2%

Abbreviations: KPS, Karnofsky Performance Status

**Table 2:** Study Measures

Outcome Measures		
Carolina Frailty Index		
Mean (SD)	0.22 (0.16)	
Range	0-0.64	
Robust (0 - <0.2)	54.10%	
Pre-frail (≥0.2 - 0.35)	22.10%	
Frail (≥ 0.35)	23.80%	
Physical function score		
Mean (SD)	12.4 (6.2)	
<20 (%)	93.20%	
IADL score		
Mean (SD)	12.5 (2.3)	
<14 (%)	42.60%	
Timed Get Up & Go Test		
Mean (SD)	12.8 (5.0)	
>=14 (% Patients)	40.60%	

Inflammatory Markers		
	Mean (SD)	Median
Total WBC (10^3^/mm^3^)	7.80 (2.93)	7.40
Neutrophils (10^3^/mm^3^)	5.48 (2.91)	4.70
Lymphocytes (10^3^/mm^3^)	1.58 (0.73)	1.50
Monocytes (10^3^/mm^3^)	0.45 (0.17)	0.40
Platelets (10^3^/mm^3^)	278.99 (99.58)	261.00
NLR	4.49 (4.19)	3.19
LMR	3.87 (2.21)	3.33
PLR	214.79 (146.96)	166.14

Abbreviations: IADL, instrumental activities of daily living; LMR, lymphocyte monocyte ratio: NLR, neutrophil lymphocyte ratio; PLR, platelet lymphocyte ratio; SD, standard deviation; WBC, white blood cell

Recently, ratios of cellular markers of inflammation such as neutrophil lymphocyte ratio (NLR), lymphocyte monocyte ratio (LMR) and platelet lymphocyte ratio (PLR) were reported to have robust prognostic value in various types of cancer [18-20]. However, the relationship of total and differential WBC counts, particularly neutrophils, monocytes and lymphocytes, with frailty have not yet been explored in older adults with cancer. In addition, the association between frailty and these novel inflammatory markers -- NLR, LMR and PLR -- has not been investigated in older patients with cancer. To investigate these research questions, we analyzed data from a registry of geriatric assessments conducted in older adults with cancer, to examine the association between frailty and total and differential WBC counts as well as ratios of cellular inflammatory makers.

### Frailty and inflammatory markers

**Figure 1:**
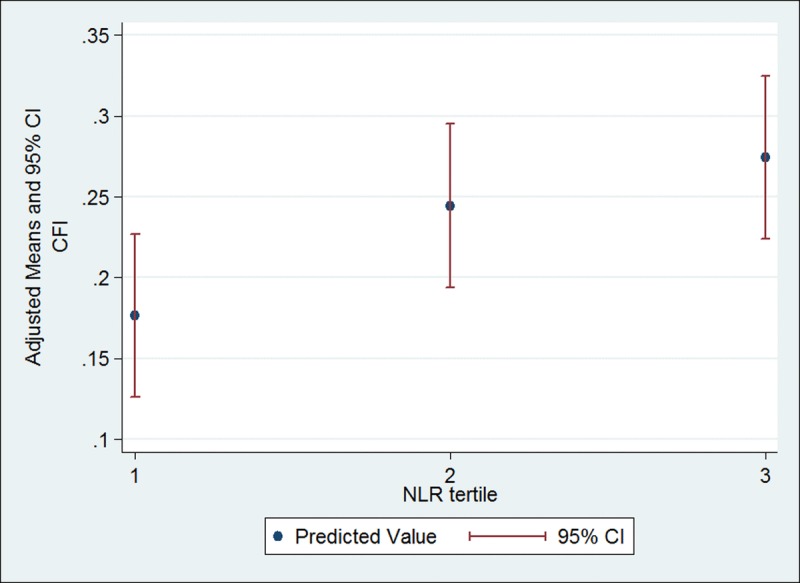
Multivariable linear regression analysis of the CFI with NLR tertile NLR tertile 1 is <2.5, NLR tertile 2 is 2.5-4.2 and NLR tertile 3 is >4.2. Multivariable linear regression was adjusted for age, sex, race, education, marital status, cancer type and cancer stage. Abbreviations: CFI, Carolina frailty index; CI, confidence interval; NLR, neutrophil lymphocyte ratio

Bivariable associations between the CFI and inflammatory markers are summarized in Table 3. NLR was positively correlated with the CFI (r = 0.220, p = 0.025) using Spearman's correlation test. There was a fair negative correlation between LMR and the CFI (r = −0.185, p = 0.062) and positive correlation between PLR and the CFI (r = 0.178, p = 0.072). We took tertiles of NLR, LMR and PLR for multivariable analyses. Cutoff values were <2.5, 2.5-4.2, and >4.2 for NLR, <2.8, 2.8-4.3, and >4.3 for LMR and <142, 142-210, and >210 for PLR. Simple and multivariable linear regression analyses of the CFI with NLR, LMR and PLR tertiles are summarized in Table 4. After adjusting for the baseline characteristics (e.g., age, sex, race, education, marital status, cancer type, and cancer stage), patients with NLR in the top tertile were significantly more frail (higher mean CFI) compared to those in the bottom tertile (Table 4). The estimated adjusted difference in the CFI between the top and bottom tertiles was 0.098. An adjusted mean CFI was 0.27 in NLR top tertile patients and 0.18 in bottom tertile patients (Figure 1). No significant difference in the CFI was observed across tertiles of LMR or PLR in the multivariable linear regression models. For ease of interpretation by clinicians, the CFI was categorized into frail or pre-frail versus robust and the association between tertiles of ratio markers and frailty status was examined using multivariable logistic regression analysis. After adjusting for the same seven variables as listed above, patients in the top tertile of NLR had increased odds of being frail/pre-frail compared with those in the bottom tertile (OR=3.81; CI, 1.13-12.84, Table 4).

**Table 3:** Bivariable associations between Carolina Frailty Index and the inflammatory markers

Carolina Frailty Index		
Inflammatory Markers	Spearman's rho	p Value
Total WBC	0.115	0.208
Neutrophils	0.163	0.100
Lymphocytes	−0.140	0.157
Monocytes	0.135	0.174
Platelets	0.114	0.213
NLR	0.220	0.025
LMR	−0.185	0.062
PLR	0.178	0.072

Abbreviations: LMR, lymphocyte monocyte ratio: NLR, neutrophil lymphocyte ratio; PLR, platelet lymphocyte ratio; WBC, white blood cell

**Table 4:** Multivariable analysis of the association between frailty and the inflammatory markers

	Simple linear regression		Multivariable linear regression		Multivariable logistic regression	
	Regression Coefficient (95% CI)	p value	Regression Coefficient (95% CI)	p value	Odds Ratio (95% CI)	p value
NLR tertile						
Bottom tertile (<2.5)	Reference		Reference		Reference	
Middle tertile (2.5-4.2)	0.059 (−0.205 to 0.138)	0.144	0.068 (−0.005 to 0.141)	0.067	2.61 (0.83 to 8.19)	0.100
Top tertile (>4.2)	0.096 (0.019 to 0.173)	0.015	0.098 (0.023 to 0.173)	0.011	3.81 (1.13 to 12.84)	0.031
LMR tertile						
Bottom tertile (<2.8)	Reference		Reference		Reference	
Middle tertile (2.8-4.3)	−0.009 (−0.089 to 0.072)	0.829	−0.001 (−0.080 to 0.078)	0.975	1.04 (0.32 to 3.40)	0.947
Top tertile (>4.3)	0.055 (−0.025 to 0.135)	0.177	0.045 (−0.035 to 0.125)	0.269	2.17 (0.65 to 7.25)	0.210
PLR tertile						
Bottom tertile (<142)	Reference		Reference		Reference	
Middle tertile (142-210)	0.061 (−0.019 to 0.141)	0.133	0.054 (−0.019 to 0.128)	0.143	2.03 (0.67 to 6.10)	0.209
Top tertile (>210)	0.064 (−0.016 to 0.143)	0.115	0.039 (−0.038 to 0.116)	0.321	1.71 (0.54 to 5.45)	0.361

Multivariable linear regression and multivariable logistic regression models were adjusted for age, sex, race, education, marital status, cancer type and cancer stage. Abbreviations: CI, confidence interval; LMR, lymphocyte monocyte ratio: NLR, neutrophil lymphocyte ratio; PLR, platelet lymphocyte ratio

### Functional GA measures and inflammatory markers

We evaluated associations between functional GA measures (physical function, IADL, and TUG) and the inflammatory markers (Table 5). Higher NLR was associated with lower IADL score (r = −0.203, p = 0.040). There was also a positive correlation between LMR and IADL score (r = 0.197, p = 0.046). Patients with a TUG score ≥ 14 had significantly higher median NLR as compared to patients with a score < 14 (p = 0.016). Median LMR was significantly lower in patients with prolonged TUG compared with those with a normal TUG score (p = 0.013).

**Table 5:** Bivariable associations between functional GA measures and the inflammatory markers

Physical function score			
Inflammatory Markers	Spearman's rho		p Value
Total WBC	−0.050		0.590
Neutrophils	−0.112		0.270
Lymphocytes	0.133		0.187
Monocytes	−0.040		0.696
Platelets	−0.142		0.124
NLR	−0.185		0.066
LMR	0.132		0.190
PLR	−0.188		0.062

IADL score			
Inflammatory Markers	Spearman's rho		p Value
Total WBC	−0.112		0.219
Neutrophils	−0.178		0.073
Lymphocytes	0.094		0.346
Monocytes	−0.187		0.058
Platelets	−0.070		0.443
NLR	−0.203		0.040
LMR	0.197		0.046
PLR	−0.120		0.227

Timed Up & Go Test			
Inflammatory Markers	TUG ≥ 14 (N=44)	TUG <14 (N=63)	p Value
Total WBC, median	7.80	7.30	0.407
Neutrophils, median	5.15	4.60	0.153
Lymphocytes, median	1.20	1.60	0.041
Monocytes, median	0.50	0.40	0.206
Platelets, median	262.00	261.00	0.700
NLR, median	3.70	2.83	0.016
LMR, median	3.00	3.75	0.013
PLR, median	189.46	154.58	0.069

Abbreviations: IADL, instrumental activities of daily living; LMR, lymphocyte monocyte ratio: NLR, neutrophil lymphocyte ratio; PLR, platelet lymphocyte ratio; TUG, Timed Up & Go Test; WBC, white blood cell

## DISCUSSION

Frailty is a syndrome affecting physiologic reserve across multiple organ systems. Longitudinal studies in geriatric populations have shown that frailty is associated with functional decline, hospitalization and death [2, 5, 21]. In geriatric oncology, Cohen et al. reported that pre-frail and frail status is associated with a higher risk of high-grade toxicity, treatment discontinuation and hospitalization compared with robust status in cancer patients aged >65 years receiving chemotherapy. They categorized the patients into robust, pre-frail and frail status using a deficit-accumulation frailty index which is a 51-item scale with geriatric assessment variables and laboratory values [22]. Recently, our group evaluated the prognostic value of the Carolina Frailty Index (CFI) in a dataset derived from the linkage of the Carolina Senior Registry, which contains GA data, and all-cause mortality data obtained from the North Carolina Central Cancer Registry. The CFI was found to be a prognostic factor for all-cause mortality in this analysis [10]. Using this validated CFI, in the study reported here, we performed a cross-sectional analysis of older adults with cancer to evaluate the association between frailty and CBC-based inflammatory markers. We found that patients with NLR in the top tertile were significantly more likely to be frail or pre-frail (OR = 3.81; 95% CI, 1.13-12.84) as compared to those with NLR in the bottom tertile, after adjusting for the potential confounders.

The importance of inflammation in frailty has been shown consistently in the general population of older adults using both Fried and Rockwood models [12, 23-25]. IL-6 is the most studied non-cellular marker of inflammation and is consistently reported to be associated with frailty in both cross-sectional and prospective studies [12, 23-26]. Recently, Brouwers et al. explored the relationship of biomarkers including IL-6 and frailty in a cross-sectional study of 82 young and 162 older (≥ 70 years) breast cancer patients. IL-6 levels were significantly different between the three Balducci frailty categories -- median values for fit, vulnerable and frail subjects were 1.4, 2.3 and 2.8 pg/ml, respectively (p = 0.019) [27]. In terms of cellular markers of inflammation, high total WBC, neutrophil and monocyte counts were associated with an increased prevalence of frailty in non-cancer geriatric populations [12,13, 25]. Interestingly, Collerton et al. reported a significant negative correlation between lymphocyte count and frailty in a cross-sectional study of persons 85 years or older (n = 845), and this association remained significant after adjusting for potential confounders [25]. Their finding suggests that immunosenescence, defined as aging-related alterations of the immune system, may be associated with frailty as a low lymphocyte count is thought to be a crude marker of immunosenescence [28]. Although no significant associations of total and differential WBC counts with frailty were observed in the present study, there was a significant association between frailty and NLR in multivariable analyses. In addition, higher NLR was significantly associated with lower IADL scores and prolonged TUG. We believe NLR is a more robust marker for frailty than neutrophil or lymphocyte count alone because it combines the two cellular markers which are associated with frailty and likely to reflect the underlying pathophysiology of frailty.

The prognostic role of NLR has been extensively studied in various cancers. Templeton et al. performed a meta-analysis to quantify the prognostic value of NLR on clinical outcome in patients with solid tumors [18]. They included 100 studies comprising 40,559 patients in their analysis. Overall, NLR greater than the cutoff was associated with a hazard ratio for poorer overall survival of 1.81 (95% CI = 1.67 to 1.97; P < 0.001). A high NLR was also associated with adverse cancer-specific, progression-free and disease-free survival. Mechanisms underlying the relationship of high NLR with poor outcomes in cancer patients are poorly understood. High NLR may reflect an inflammatory state where the cytotoxic activity of immune cells such as activated T cells and natural killer cells is suppressed by inflammatory cytokines produced by neutrophils [29, 30].

Our study had some limitations. First, the sample size for this study was relatively small and provided limited statistical power. Second, we had some missing variables in the section of the GA completed by the patient. Missing variables are taken into account when calculating the CFI by subtracting a number of missing variables from the denominator. Based on the previous work of Rockwood et al. [31], we believe the frailty index calculated this way gives a reasonable assessment of frailty status. However, there were two patients who missed more variables (13 and 15 variables) than other patients and we performed a sensitivity analysis by removing these two patients from the multivariable logistic regression model. In this analysis, patients in the top tertile of NLR had an odds ratio of 4.1 (95% CI = 1.2-14.6) for frail/pre-frail status. This result is similar to the result with the entire sample (odds ratio = 3.8; 95% CI = 1.1-12.8). Third, we could not assess the causality of the identified associations between frailty and inflammatory markers in this cross-sectional study. Longitudinal studies are warranted to investigate the predictive effects of these markers on frailty. Forth, although patients with clinical evidence of acute infection were excluded from our study, we could not completely eliminate the possibility that subclinical infections may have affected total and differential WBC counts in some patients in our sample. Finally, the largest proportion of patients in our sample was breast cancer patients and most were non-Hispanic white. This limits the generalizability of our results to the general population of cancer patients. However, our findings are consistent with findings from large population-based studies conducted in geriatric populations.

In conclusion, this study provides further evidence linking frailty and inflammatory markers in older adults with cancer. As NLR is a simple and readily available inflammatory marker associated with frailty and survival outcomes in cancer patients, it warrants further investigation in larger studies with a special focus on their utility in clinical practice. NLR may be a useful marker that could help identify older adults with cancer who will benefit from the evaluation of frailty status with geriatric assessment.

## METHODS

### Study design and patient population

This study is a cross-sectional analysis of data from the “Carolina Senior: Registry for Older Patients” (CSR; ClinicalTrials.gov identifier NCT01137825). CSR is a database of English-speaking cancer patients 65 years or older who completed a brief GA [11, 32, 33]. Participants were recruited from oncology clinics at the North Carolina Cancer Hospital and community clinics across the state [34]. Informed consent was obtained from all patients prior to participation. The current secondary data analysis was limited to patients with data on pretreatment GA and complete blood count (CBC) with or without differential. Patients with acute infection at the time of baseline CBC test, leukemia, or a history of previous chemotherapy, radiotherapy or stem cell transplant were excluded. The study protocol was approved by the UNC Institutional Review Board.

### Study measures

#### Geriatric assessment (GA)

The GA used in the CSR was developed by Hurria et al. [11] and is comprised of validated measures, some of which are completed by a health care professional or research assistant and the remainder by a patient [32,33]. The section of the GA completed by a health-care professional includes the following measures: Timed Up and Go (TUG) test [35], Karnofsky Performance Status (KPS) [36], Blessed Orientation Memory Concentration (BOMC) test [37], and Body Mass Index (BMI) [38]. Measures that are completed by a patient include instrumental activities of daily living (IADL) [39], physical function [40], patient-reported KPS [41], vision [42], hearing [43], medications [44], comorbidities [45], nutritional status [46], mental health [47], and falls.

#### Frailty index

The primary outcome in our study is frailty, as determined by the 36-item Carolina Frailty Index (CFI) [10]. The cancer-specific frailty index was originally devised using 32 GA variables [9] following the methodology reported in Searle and Rockwood et al. [48]. Recently, the 32-item frailty index was revised by adding four more variables and named the Carolina Frailty Index. The CFI was associated with all-cause mortality in elderly cancer patients [10]. The CFI is a continuous variable with range 0–1 and expressed as a ratio of deficits present to the total number of deficit variables with completed data. Total deficits were calculated by summing them (0 = absence of deficit and 1 = presence of deficit). For example, if 36 deficit variables are available (i.e. none missing), and 10 deficits are identified in a patient, that person's frailty index would be 10/36 = 0.28. Using the CFI, Guerard et al. categorizes older persons as robust (0 to <0.2), pre-frail (0.2 to < 0.35), or frail (≥ 0.35) [48]. GA domains included in the CFI are physical function, instrumental activities of daily living (IADL), timed up and go (TUG), vision, hearing, falls, comorbidities, medications, nutritional status, cognitive function, mental health, and social activity. A list of all variables in the index and cut points for frailty status are shown in Supplement 1. Study subjects were included if they had data on at least half of the 36 variables. This is based on the work by Rockwood et al. showing that the results yielded by the frailty index have been consistent between studies that consider different deficits, or different number of deficits provided they adequately cover the important geriatric domains [3,4,31]. For the CFI, 59 % of patients had no missing data, 39% had missing data on 1-4 variables, and two patients missed more than 4 variables (they missed 13 and 15 variables, respectively).

#### Functional GA measures

Secondary outcomes for our study are GA function measures such as physical function, IADL, and Timed Up and Go (TUG) test. The physical function scale is a subscale of the Medical Outcomes Study (MOS) Physical Health. It measures limitations in engaging in various activities ranging from “bathing and dressing?” to “vigorous activities, such as running or lifting heavy objects”, with response options of 2 (“not at all limited”), 1 (“limited a little”), and 0 (“limited a lot”) [39]. Scores range from 0 to 20, with lower scores signifying dependence in performing the activities. The IADL scale measures the need for assistance with using the telephone, certain modes of transportation, shopping, food preparation, housekeeping, taking medications, and handling finances [40]. It is formatted for self-administration and uses a 3-point Likert-type scale (0=totally dependent, 1=partially dependent, 2=totally autonomous), total score range of 0 to 14. The TUG test asks the patient to stand up from a chair, walk a distance of approximately 10 feet, turn, walk back to the chair, and sit down; total seconds required to complete the test are recorded or “inability to complete” is noted [35].

#### Inflammatory markersn

Pretreatment total and differential WBC and platelet counts were abstracted from medical records. Total WBC, neutrophil, monocyte, lymphocyte, and platelet counts and neutrophil lymphocyte ratio (NLR), lymphocyte monocyte ratio (LMR) and platelet lymphocyte ratio (PLR) were assessed as the independent variables.

### Statistical analysis

Descriptive statistics were used to describe baseline characteristics of the sample. The primary outcome variable was the CFI score. Secondary outcome variables were physical function, IADL, and TUG. We examined bivariable associations between the outcome variables (CFI, physical function, and IADL) and each of the inflammatory markers (total WBC, neutrophil, monocyte, lymphocyte, and platelet counts, NLR, LMR and PLR) using Spearman's correlation test. TUG score was dichotomized at a cut point of 14, which is predictive of falls [49], and the Wilcoxon Rank-sum test was used to assess the bivariable association between TUG and the inflammatory markers. Multivariable linear and logistic regression models were used to assess the independent effects of inflammatory markers on frailty status. Due to non-normal distributions, the inflammatory markers were modeled as tertiles for their association with frailty. To maximize statistical power, the CFI was initially analyzed as continuous outcome variable in the linear regression models. Then, the CFI was categorized into frail or pre-frail versus robust status for more straightforward clinical interpretation and to calculate odds ratios using logistic regression models. Covariates were age (continuous variable), sex, race (white vs non-white), education (< high school vs ≥ high school graduate), marital status (married vs unmarried), cancer type (breast vs other cancer), and cancer stage (stage IV vs I, II and III). MOS physical function, IADL, TUG and KPS were not included as covariates in the multivariable analyses because these measures were used as variables in the CFI. SAS statistical software version 9.3 (SAS Institute Inc., Cary, NC) and Stata 14 software (College Station, TX: StataCorp LP) were used for analyses.

## SUPPLEMENTARY MATERIALS TABLE


