# Supplementary material for: Frailty and inflammatory markers in older adults with cancer
Source: Aging (Albany NY). 2017 Mar 8;9(3):650–61. doi: 10.18632/aging.101162 (PMC5391224; doi:10.18632/aging.101162)
Supplement: Supplementary file 1 [file aging-09-650-s001.pdf]

SUPPLEMENTARY MATERIAL

Supplementary Table S1. Carolina Frailty Index Variables.

Item	Format	Score
**Can you use a telephone**	Without help<br>With some help<br>Unable	0<br>1<br>1
**Can you get to places out of walking distance**	Without help<br>With some help<br>Unable	0<br>1<br>1
**Can you go shopping for groceries or clothes**	Without help<br>With some help<br>Unable	0<br>1<br>1
**Can you prepare your own meals**	Without help<br>With some help<br>Unable	0<br>1<br>1
**Can you do housework**	Without help<br>With some help<br>Unable	0<br>1<br>1
**Can you take your own medicines**	Without help<br>With some help<br>Unable	0<br>1<br>1
**Can you handle your own money**	Without help<br>With some help<br>Unable	0<br>1<br>1
**Lifting or carrying groceries**	Not limited at all<br>Limited a little<br>Limited a lot	0<br>1<br>1
**Climbing one flight of stairs**	Not limited at all<br>Limited a little<br>Limited a lot	0<br>1<br>1
**Bending, kneeling, or stooping**	Not limited at all<br>Limited a little<br>Limited a lot	0<br>1<br>1
**Walking one block**	Not limited at all<br>Limited a little<br>Limited a lot	0<br>1<br>1
**Bathing or dressing yourself**	Not limited at all<br>Limited a little<br>Limited a lot	0<br>1<br>1
**Self reported Health**	Normal<br>Minor symptoms of disease<br>Some symptoms of disease<br>Care for self only<br>require occasional assistance<br>require considerable assistance<br>disable<br>severely disabled	0<br>0<br>0.5<br>1<br>1<br>1<br>1<br>1
**Falls in the last 6 months**	0<br>1 or more	0<br>1
**Number of Daily Medications**	0-8<br>>9	0<br>1
**Other cancers or leukemia**	No<br>Yes	0<br>1
**Arthritis or Rheumatism**	No<br>Yes	0<br>1

Item	Format	Score
**Glaucoma**	No<br>Yes	0<br>1
**Emphysema or chronic bronchitis**	No<br>Yes	0<br>1
**High blood pressure**	No<br>Yes	0<br>1
**Heart Disease**	No<br>Yes	0<br>1
**Circulation trouble in arms or legs**	No<br>Yes	0<br>1
**Diabetes**	No<br>Yes	0<br>1
**Stomach or intestinal disorders**	No<br>Yes	0<br>1
**Osteoporosis**	No<br>Yes	0<br>1
**Chronic liver or kidney disease**	No<br>Yes	0<br>1
**Stroke**	No<br>Yes	0<br>1
**Depression**	No<br>Yes	0<br>1
**Hearing**	Excellent<br>Good<br>Fair<br>Poor<br>Totally Deaf	0<br>0<br>1<br>1<br>1
**Vision**	Excellent<br>Good<br>Fair<br>Poor<br>Totally Blind	0<br>0<br>1<br>1<br>1
**Unintentional Weight Loss**	No<br>Yes < 5%<br>Yes > 5%	0<br>0<br>1
**Felt downhearted or blue**	None of time<br>A little of the time<br>Some of the time<br>A good bit of time<br>Most of the time<br>All of the time	0<br>0<br>0.5<br>1<br>1<br>1
**Felt calm or peaceful**	All of the time<br>Most of the time<br>A good bit of time<br>Some of the time<br>A little of the time<br>None of the time	0<br>0<br>0<br>0.5<br>1<br>1
**Physical or emotional health interfere with social activities**	None of time<br>A little of the time<br>Some of the time<br>Most of the time<br>All of the time	0<br>0<br>0.5<br>1<br>1
**Blessed-Orientation-Memory-Concentration**	Less than 11<br>11 or greater	0<br>1

Item	Format	Score
**Timed up and go**	Less than 14<br>14 or greater	0<br>1
**Total Number of Items = 36**		

Coding of Variables is as follows:

- "0" = absence of deficit
- "1" = presence of deficit
- "0.5" is used for intermediate responses
- Add up scores for each patient and divide by number of variables to achieve a frailty index score
